# Social capital in a regional inter-hospital network among trauma centers (trauma network): results of a qualitative study in Germany

**DOI:** 10.1186/s12913-018-2918-z

**Published:** 2018-02-26

**Authors:** Julika Loss, Johannes Weigl, Antonio Ernstberger, Michael Nerlich, Michael Koller, Janina Curbach

**Affiliations:** 1 Medical Sociology, University of Regensburg, Dr Gessler-Str. 17, D-93049 Regensburg, Germany; 2 Department of Trauma Surgery, University Medical Center, 93053 Regensburg, Germany; 3 Center for Clinical Studies, University Medical Center, Regensburg, Germany

**Keywords:** Germany, Social capital, Trauma network, Multiple trauma, Inter-hospital network, Cooperation, Hierarchy, Leadership style

## Abstract

**Background:**

As inter-hospital alliances have become increasingly popular in the healthcare sector, it is important to understand the challenges and benefits that the interaction between representatives of different hospitals entail. A prominent example of inter-hospital alliances are certified ‘trauma networks’, which consist of 5-30 trauma departments in a given region. Trauma networks are designed to improve trauma care by providing a coordinated response to injury, and have developed across the USA and multiple European countries since the 1960s. Their members need to interact regularly, e.g. develop joint protocols for patient transfer, or discuss patient safety. Social capital is a concept focusing on the development and benefits of relations and interactions within a network. The aim of our study was to explore how social capital is generated and used in a regional German trauma network.

**Methods:**

In this qualitative study, we performed semi-standardized face-to-face interviews with 23 senior trauma surgeons (2013-14). They were the official representatives of 23 out of 26 member hospitals of the Trauma Network Eastern Bavaria. The interviews covered the structure and functioning of the network, climate and reciprocity within the network, the development of social identity, and different resources and benefits derived from the network (e.g. facilitation of interactions, advocacy, work satisfaction). Transcripts were coded using thematic content analysis.

**Results:**

According to the interviews, the studied trauma network became a group of surgeons with substantial bonding social capital. The surgeons perceived that the network’s culture of interaction was flat, and they identified with the network due to a climate of mutual respect. They felt that the inclusive leadership helped establish a norm of reciprocity. Among the interviewed surgeons, the gain of technical information was seen as less important than the exchange of information on political aspects. The perceived resources derived from this social capital were smoother interactions, a higher medical credibility, and joint advocacy securing certain privileges.

**Conclusion:**

Apart from addressing quality of care, a trauma network may, by way of strengthening social capital among its members, serve as a valuable resource for the participating surgeons. Some member hospitals could exploit the social capital for strategic benefits.

## Background

Inter-hospital alliances have become increasingly popular in the contemporary healthcare sector. Examples include partnerships between academic medical centers and community hospitals, networks of rural hospitals in a region, and collaborations of university hospitals dedicated to highly specialized procedures [1–4]. The increase of alliances has been explained by the hospitals’ need to control costs, to improve patient accessibility and to foster efficiency and continuity of care, and to form a united front for advocacy [3, 5–7]. A major attraction of networks for providers – as opposed to full-asset mergers – is the ability to maintain local autonomy while increasing their power as part of a group [1, 8]. Opportunities offered by hospital alliances include shared learning and access to expertise, thus supporting the spreading of innovations and adoption of new clinical practices [9]. Banding together with other hospitals can also strengthen an organization’s market position, by creating an impression of size and strength, and increase its power [8, 9]; competitive efforts can be directed towards others outside the network instead of against each other [9]. A network of healthcare organizations in a geographical area can also choose to devote itself to maximize the customer’s utility (e.g. speed of access to medical care) rather than private profit [10]; multi-stakeholder community partnerships can coordinate patient care across the continuum of care and thus decrease problems of fragmentation [11]. Hospital alliances can be challenging, however, mainly due to role ambiguities or conflicting interests, as the network members may be situated in a competitive environment or represent different healthcare sectors [11, 12]. In addition, hospital representatives may need numerous sessions to align their interests and decide upon the balance of what will be shared and what is proprietary, and efforts may be required to maintain the cooperation over time [9, 11].

A prominent example of inter-hospital networks are the regional ‘trauma networks’, or ‘trauma systems’, which consist of trauma care facilities of all levels in a given region. Trauma networks are designed to improve trauma care by providing a coordinated response to injury. These inter-hospital networks have developed across multiple European countries, e.g. England [13], France [14], Switzerland, or the Netherlands [15]. The idea of an organized approach to severe trauma care dates back to the 1960s when the first statewide trauma system was established in Maryland, USA [15, 16]. Fundamental to the trauma system infrastructure is a network of hospitals committed to high-quality, standardized treatment of individuals with injuries [17], with the aim of admitting patients with severe trauma to the most suitable trauma center [14]. The trauma networks do not evolve informally, but are set up, orchestrated and accredited by a country’s responsible medical trauma association. They have a ‘lead hospital’ or coordinator, which usually represents the highest level of care available within the regional system [18]. An essential part of a trauma system is the development of mutually agreed-upon written protocols for the transfer of patients between the cooperating institutions. In addition, the participating hospitals are required to exchange expertise through training programs, discuss patient safety issues between hospitals, and harmonize clinical practice guidelines [18, 19]. Several studies have shown the survival benefit of treating trauma patients within a trauma network and in specialized centers. In Germany, the German Trauma Society initiated the establishment of regional trauma networks (“TraumaNetzwerk DGU®”) in 2006 [20]. Here, auditors visit trauma units of hospitals and certify them either as local, regional, or supra-regional trauma center, according to the respective personnel and structural requirements they comply with. The trauma units in a German region are organized in a network of a minimum of five trauma centers, including preferably at least one supra-regional center. They are required to develop a contract of cooperation consisting of consented written guidelines on inter-hospital patient transfer, regular joint meetings (quality circles) and continuing education [20]. By September 2014, a total of 49 trauma networks had been successfully certified in Germany.

From a social science perspective, a trauma network can be viewed as a group of individual senior medical officers (clinical directors) drawn from across different hospitals, geographic areas and levels of care, who meet on a regular basis, interact, and agree upon decisions, e.g. in terms of patient transfer, quality improvement, and standardization. Apart from the official standards for cooperation, informal social network properties such as leadership style, information flow, mutual trust, and shared goals can be held necessary for their effective collaboration. These dimensions of network interactions are well captured in the concept of social capital. Social capital is embedded in relations between individuals [21], and can be defined as the sum of resources attainable by individuals, groups, organizations, and communities through a durable network of social relationships [21–23].

Social capital is constituted by a network’s shared values and norms, and reciprocity and trust within the network [22]. The concept of social capital has been put forward by different authors, above all Bourdieu Alone, Coleman and Putnam. Although these authors share a common core idea of social capital, i.e. social relations holding resources for the individuals who are part of a group or network, they differ in terms of the way individuals or groups can activate these resources, and in terms of the value that social capital has e.g. on the level of the society. While Bourdieu stresses the role of social capital as limited resource of individuals who strive to maintain and reproduce collective (upper) class privileges, identity and membership [23], Putnam [24] and Coleman [21] focus on how social capital in organizations, networks and communities can increase trust, facilitate inter-personal cooperation and the pursuit of shared goals. It is argued that social capital and its relational, shared norms facilitate cooperation and may reduce transaction costs and conflict in interactions [21, 24, 25]. In sum, social capital is useful to solve collective problems and to enhance the outcomes of purposive action, which is interesting especially with regard to the tasks of trauma networks.

In addition, the economic literature draws on social capital resources to be valuable for a company’s strategy, e.g. by allowing the gain of (technical) information and the building of strategic alliances [26, 27]. In the case of hospital networks, the economic view may be relevant as well, because ties to other hospitals may provide a trauma unit with knowledge to improve patient care and thus contribute to the economic success of the clinic.

Adding to this, the literature identifies three different forms of social capital: bonding, bridging and linking social capital. Bonding social capital relates to strong ties among homogeneous groups and can give these particular groups a sense of identity and common purpose. Bridging social capital refers to relations within socially heterogeneous groups by weaker, but more cross-cutting ties. Bridging ties are less effective in generating trust and norms, but can create open-mindedness and integration of marginalized groups, and the bridging of disconnected groups can make new information available [26, 28, 29]. Linking social capital is characterized by relations between those people within a hierarchy where there are differing levels of power [30].

Reflecting the diversity of perspectives on social capital, a variety of sets of indicators have been put forward for its measurement [31–35]. They have in common that they capture information on a group’s or network’s personal relations (knowledge of personal information), its structure and functioning (e.g. frequency of coming together, hierarchy), on its social climate (e.g. trust, reciprocity) and its identity (e.g. shared values, goals). In addition, different categories are proposed to describe resources and benefits that are generated by the interactions, e.g. knowledge resources (being well informed about the other members), advocacy and power (e.g. collective actions, influence in local, regional or national affairs), health and life satisfaction, and status. The activation of resources can refer to the individual who is part of the group, or the group as a whole (collective benefits).

Most previous studies have focused on social capital in communities, neighborhoods and geographical areas; some studies have investigated the role of social capital in the health care setting. These studies have mainly focused on social capital of physicians or health professionals generated via teamwork within their respective workplaces, e.g. hospitals or community health centers [34, 36–38]. Some studies have taken an explicit interest in how social capital is associated with the coordination of professional hospital staff and hospital organizational effectiveness [39–41]. On the individual level, physician social capital has been analyzed regarding the access to and uptake of medical knowledge [42, 43].

We did not find any studies, however, analyzing if and how social capital can be generated among a group of physicians who co-operate in an officially set-up inter-hospital network, and how social capital resources can be activated for the individual or mutual benefit. The current study thus differs from pre-existing social capital studies in that its focus is not on social ties in a single workplace setting, but intends to analyze the role of social capital in a cross-organizational network of healthcare professionals. It is also worth remarking that most healthcare alliances that have been studied before consist of multi-stakeholder community networks between different healthcare sectors across the continuum of care (e.g. primary care – hospital – rehab, see also [11]). Our study also differs from available studies on healthcare alliances by applying social capital theory in the analysis of cooperative relations and processes. Apart from this, our research interest is not to analyze the trauma network’s effectiveness in terms of its formal tasks and goals, but to analyze the informal social contexts that may play a role in how members engage in network interaction and what they perceive to be the benefits and implications of this interaction.

The aim of the present research is to use the case of a long-standing regional trauma network (TraumaNetzwerk DGU® Eastern Bavaria, TNO) in order to explore

- how social capital is generated and maintained in a regional co-operative network of trauma clinics, and the factors that influence the development of social capital, as viewed by the network’s members
- the potential collective and individual benefits that members attribute to the trauma network’s social capital

In specific, we apply social capital theory to analyze the social aspects of the trauma network. The specific case was chosen because it had been the first German trauma network to be certified, and it was also one of the largest trauma networks, thus offering the potential to collect rich data material.

Based on the concepts and indicators of social capital theory as described above, we raise following assumptions:

1. *Relations:* Being organized in the trauma network requires regular meetings, joint decision-making, and co-operation among its participants. We therefore assumed that personal relations between the individuals will develop and intensify. Relation would probably commence with learning who the other trauma networks members are, where they are affiliated, and what interests and expertise they have. This shared knowledge of personal information can serve as knowledge resource (see also [32]).
2. *Structure and functioning:* We assumed that the way the social relations and interactions develop depends on the network’s structure and functioning. This includes the frequency of the trauma network’s members coming together, and the way their interaction and co-operation is organized and facilitated. We assumed that hierarchy may play an important role, as the different levels of trauma care included (local, regional, supra-regional) may be reflected in the network’s interactions. We also assumed that the way that the hierarchical differences are dealt with is important for the development of the group’s social capital. In addition, norms of respect and trust between people who are interacting across power or authority gradients (“linking social capital”) may be relevant in this context [28].
3. *Trust and reciprocity:* We assumed that substantial social capital can only be generated in the studied trauma network when the interactions are characterized by trust and reciprocity [22, 28, 31]. The network consists of a very homogeneous group of trauma surgeons, a fact which may facilitate the understanding of each other’s concerns, empathy and readiness to be supportive (“bonding social capital” [28]). On the other hand, the members of the trauma network are potential competitors, which may be a barrier for the development of trust and solidarity.
4. *Shared goal:* We assumed that the trauma network members were united by a shared goal (e.g. to improve the care of trauma patients), which is a key element of social capital [22]. However, the agreement on a shared goal could not be taken for granted, considering that the studied trauma network has not (merely) formed ‘bottom up’ by interested trauma surgeons, but was also officially demanded by the German Trauma Society. We also assumed that the trauma network could be a platform in which professional goals could be pursued in a joint endeavor and via joint activities.
5. *Benefits on the network level:* Social capital theory draws on the conviction that social relations hold resources for the individuals who are part of a group. We assumed that the studied trauma network may be able to utilize any social capital that may arise from their interactions in a collective way, e.g. by gaining power as a group, or by improving the trauma care in their region. We assumed that the individual members may be also able to activate social capital resources, should they occur, for their own benefit, e.g. by facilitating inter-personal communication and reducing conflict [21, 24, 25] in situations of patient transferals. As social capital within health care organizations has been linked to job satisfaction of health professionals [34], we assumed that social capital of the trauma network may also influence how content its members are with certain aspects of their clinical work.
6. *Benefits on the hospital level (strategic benefits):* In the economic literature, social capital resources are valuable because they can be utilized to gain access to (technical) information and expertise [26, 27]. In the case of the trauma network, we assumed that ties to other hospitals may provide a trauma unit with knowledge to improve patient care and thus contribute to the clinical outcome as well as economic success of the clinic.

## Methods

### The organizational setting

The trauma network Eastern Bavaria (TNO) was certified by the German Trauma Society in 2009, after 2 years of preparations and regular meetings between the hospitals [44]. It was the first of its kind to be certified in Germany; with 26 participating hospitals, it is also one of the largest German trauma networks. The TNO now covers a catchment area of around 20,000 km^2^, which is roughly the size of Israel or Slovenia. Currently, the TNO consists of two supra-regional trauma centers (level I), both of them located in the city of Regensburg (150,000 inhabitants), nine regional (level II) and 15 local (level III) trauma centers. The Regensburg university hospital was elected as the TNO ‘leading hospital’. The present study is part of a larger research project aiming to evaluate the performance of the TNO, the study protocol of which has been published elsewhere [45].

### Data collection

In order to gain rich data for our trauma network case study, we aimed to conduct semi-structured, face-to-face interviews with the respective representatives of all trauma clinics belonging to the Trauma Network Eastern Bavaria. Of the 26 TNO hospitals, three TNO representatives refused to give an interview. The reason given for the refusals were ‘no interest in scientific studies’ (*n* = 1) and ‘a low number of multiple trauma patients treated’ (*n* = 2). Interviews were hence performed with 23 (assistant) medical directors of 23 TNO trauma clinics from 09/2013 to 09/2014. All participants (100% male) were the official trauma network representatives of their respective hospital. Four of those representatives preferred to be joined by a colleague who was believed to be able to also contribute substantially to the interview topics. Numbers and constellations of all interviews are detailed in Table 1.

**Table 1:** Interview partners in the 26 TNO hospitals

Interview partners	local trauma centre (level III) (n)	regional trauma centre (level II) (n)	supra-regional trauma centre (level I) (n)	Total (n)
Participating trauma clinics				
Medical director (senior consultant)	6	6	1	**13**
Assistant medical director (responsible consultant)	5		1	**6**
Medical director & Assistant medical director	1	2		**3**
Assistant medical director & registrar	1			**1**
	**13**	**8**	**2**	**23**
Non-participating trauma clinics	2	1		3

The boldface numbers are the added up sums of individuals

All participants approved to being interviewed and recorded. To ensure confidentiality of information, only one researcher was involved in the collection and transcription of data (JW). The data material was anonymized after transcription. The analysis and presentation of data did not allow for the identification of individual participants.

The interview guide (Table 2) covered different dimensions of social capital. For structural aspects of the network (#1 of interview guide), we drew on the classification developed by Krishna & Shrader for the World Bank [35]. Aspects of climate and trust (#2) and of social identity (#3) were detailed according to indicators proposed by Foxton & Jones for the British Office for National Statistics [33] and by Falk & Harrison, who focused specifically on interactive learning processes within groups [32]. The latter authors have also proposed indicators for knowledge resources (i.e. knowing the other network members and their expertise) which were taken up in the interview guide as well (#4). Collective action and influence on policy processes were named as benefits of social capital by most authors; therefore, these aspects were covered in the interviews as well (#5). We also included indicators used by Ommen et al., who studied social capital in German hospitals [34], in order to capture the potential profits gained for hospital work and patient care (#6). The interview guide was modified very slightly after the first two interviews, i.e. the order of questions was slightly changed, and a question on “shared values” (part of #3) was omitted, because both interview partners were not clear about what ‘values’ meant in the given context. In the actual interview situation, the order and wording of the question could slightly vary from the interview guide to adapt the questions to the context and the statements of the interviewees.

**Table 2:** Interview guide

#1. Structure, organization, functioning
✓ How frequently are you in contact with other TNO members? How often does the TNO meet as a group? ✓ Can you describe the organizational structure of the TNO: is it rather hierarchically or horizontally organized? ✓ Do you know whom to turn to in difficult situations? Have you already made use of this knowledge? ✓ Could you describe how decisions are made within the trauma network? Are the decision-making processes usually collective, are they transparent? ✓ What role does the leader of the TNO play in the network’s structure?
#2. Climate, reciprocity, trust
✓ Do you trust the other TNO members? ✓ In the group of TNO members, are you supportive of each other? Have you already done a favor to other TNO members, and vice versa? Is there something like solidarity within the TNO? ✓ Can you speak openly about problems? Are dissenting contributions and discussions valued?
#3. Social identity
✓ Do you have ideas and perspectives similar to other TNO members? ✓ Are you pursuing the same goals?
#4. Resources & benefits 1: Knowledge resources: Knowing each other
✓ By being part of the Trauma Network Eastern Bavaria (TNO), do you know more trauma surgeons from other hospitals than before? ✓ Does the TNO help you appraise your colleagues’ expertise and their personal qualities better?
#5. Resources & benefits 2: Facilitation of interactions, collective activities, advocacy, power
✓ How has participating in the trauma network changed the co-operation with the other TNO hospitals? *(prompts: communication, consultation, training, patient transfers)* ✓ Are you able to influence certain events with the support of the other TNO members? E.g. influence policy makers? ✓ Have you already been involved in common activities of the TNO? Have you jointly advocated for your interests? ✓ Were you able to achieve a goal by collectively getting active?
#6. Resources & benefits 3: Outcomes on patient care and work satisfaction
✓ In your opinion, does the cooperation in the TNO influence the quality of patient care? ✓ In your opinion, has the cooperation in the TNO an effect on your hospital’s success? ✓ Has being part of the TNO influenced your motivation to work?

One of the authors (JW) conducted all the interviews, each of which was audio-taped and on average lasted for 35 min (17-63 min).

The ethics committee of the University of Regensburg was consulted and decided that there were no ethical concerns that would require further authorized approval processes if the data material were anonymized before analysis (ref no 14-160-0162).

### Data analysis

Interview tapes were transcribed verbatim. The transcripts were classified according to the certified level of the hospital (l = local, r = regional, sr = supra-regional), anonymized and continuously numbered (Interview Partner = IP 01-IP 23), so none of the researchers, except the interviewer, could link the answers back to the interviewed surgeon or the respective trauma center. The transcripts were examined using thematic content analysis [46]. Themes were identified using a grounded conceptualization process [47]. The cognitive interest of analysis was to find out on the interviewees’ view on the characteristics of the trauma network with regard to social capital. Interview statements were thus taken at face value, data was ordered to overarching themes as laid out in the assumptions in the introduction above, while analysis was at the same time open to new, upcoming topics within and beyond these themes. Transcripts were repeatedly read before and after coding to ensure proper categorization of data. Categories were initially developed in line with the main domains covered in the interviews, but they evolved following a more detailed reading of information under each main category to identify emergent themes and subthemes.

Although the study had a cross-sectional design, the statements of the interviewees included retrospective reports (e.g. on how certain patterns of interaction had evolved over time), or explanations about causal relationships (e.g. on which factors influenced the development of a positive climate). These data were used to trace how social capital had been generated in the TNO in the view of its members, thus implying a process perspective to some extent.

To enhance the validity of the findings, the interview transcripts were read and coded independently by two authors (JW and JL); deviant findings were discussed between the two researchers and contradictory data was analyzed with particular attention [48]. To assess the validity and credibility of the findings, the results of the analysis were presented to a meeting of TNO members in May 2015, which 10 out of 23 interviewees attended. The study participants’ reactions to the analysis were then incorporated into the findings, a technique known as respondent validation [48].

## Results

The interviewed network participants perceived that the climate of respect and solidarity within the trauma network was closely linked to the network’s flat organizational culture and individuals’ identification with the network, and to individual and mutual benefits derived from the network. According to the interviewees, an empathic leadership style strongly influenced the development of social capital within the TNO. Reported benefits of the network’s social capital referred to smoother interactions within the network, but also to an improved status outside the network (e.g. credibility among patients), or maintenance of privileges (e.g. certification status). Network members felt that the leader, who was well connected with different executive boards in trauma care, could broker the group’s interests to decision makers.

The following paragraphs explain in more detail how the different dimensions of social capital were perceived and utilized by the TNO members.

1. Relations: The trauma network meetings improved personal contacts among the regional trauma surgeons

The quarterly quality circle (‘TNO meeting’), which is attended by many representatives of the TNO, is described to be the main platform for interaction within the TNO. According to the interviewees, these meetings have intensified former bilateral working relationships between some of the participants, as well as established new personal contacts to trauma surgeons, especially to those of smaller or more marginalized hospitals.

*“You get to know each other better, especially from some rural areas (…) – you would go there for a holiday, but you wouldn’t have personal contacts into the trauma department – from there, you get to know more people [in the TNO]. You get better connected.” (IP 13 l).*

Interview partners reported that they built up personal relationships with colleagues who had only been known to them by name before, and that they could now associate with their special competencies and responsibilities. Four out of twenty-three interview partners did not report to have more frequent or more intense contacts with other TNO members, but these surgeons pointed out that they had known many of the members before anyway, or that they had organizational difficulties in attending the quality circle meetings regularly, or found that these meetings offered only little opportunity for substantial personal exchange among the members.

2. Structure and functioning: The hierarchical structure of hospital levels exists, but mutual respect and transparent decision-making processes lead to the culture of flat organization

Twenty-one out of twenty-three interview partners agreed that the organizational culture of the group was flat. This was mainly attributed to the leadership style of the lead hospital’s representatives. Many interview partners emphasized that they felt treated as equals, ‘on a par’, whereas some pointed out the benevolent ‘paternal’ support of the lead hospital.

*[The TNO] is very … horizontal. And what’s very pleasant for somebody coming from a smaller hospital: you’re not being derided from above, nothing like, ‘There’s the hillbilly dork again.’ (IP 13 l)*

Nonetheless, it was well accepted that the local hospitals were naturally lower ranked than regional and supra-regional hospitals, and that the medical director of the University’s trauma department (the appointed leader of the trauma network) had a prominent influence on organizational decisions.

*[The smaller hospitals] are certainly not equal … The Uni hospital sets the agenda, and has also shown a bigger commitment. But it’s not an autocratic system. Sure, it’s a hierarchical system, and it’s good that it is, otherwise it wouldn’t work, but it is no autocratic system. (IP 20 l)*

*It’s not the case that the leader of the network …acts up, like “I am the determiner here”, no. In fact, there’s parity, we are on equal terms; although of course you see that, in the discussions, those who come from local hospitals are not matching up to the level of the regional hospitals, and the regional hospitals not to the level of the supra-regional ones.(IP 07 r)*

The described hierarchical elements were agreeable to the group because of the leadership-style of the lead hospital’s representative, which was described to be empathetic, collegial, and respectful, especially with regard to the local hospitals. The network members felt that the coordinator of the network had due regard for their respective competences and did not curtail their autonomy, which was important for the acceptance of the network.

*He [lead hospital’s representative] is very inclusive, very integrative… (…) There’s more or less only people on the top (of the TNO) who are not on their high horse… He’s doing that very well. (IP 17 sr)*

The majority of interview partners rated the decision-making processes to be transparent. Nine out of twenty-three interviewees explicitly pointed out that they felt included in the decision-making processes, and that decisions usually were collective decisions.

*Most of all decisions that we’ve had to make so far were related to the complete (…) trauma network, so it was basically a collective discussion. I’ve never felt that one is somehow pushed into one direction. (IP16 r)*

3. Trust and reciprocity: Network climate helps reveal common problems and supports bonds and a collective identity within the group

The empathic and respectful leadership style was directly translating into the climate of appreciation within the network, which, according to the interview partners, was characterized by open discourse and trust.

*We know each other (…) and we have confidence [in each other]. That it has been betrayed so far, in any way? That somebody has been played off against somebody else? I don’t believe that at all. No, quite the contrary, I think there is a solid ground for communicating openly with each other. (IP 23 l)*

Likewise, the fact that the supra-regional hospitals appreciated the achievements of the local hospitals, and took their concerns seriously, was rated to be essential for the functioning of the trauma network, by representatives of all levels of care.

*I consider [the TNO] very collegial, and I believe that the trauma network Eastern Bavaria is very sympathetic with the smaller hospitals (…). You’re not treated in a derogative way, or en passant, but you’re taken seriously. (IP 11 l)*

Almost all interviewed members (and from all hospital levels) were confident to reveal their own medical or administrative difficulties in front of the others, as well as in bilateral telephone consultations, which would help solve these problems. Some interviewees pointed out that disagreement and criticism were welcome in the discussions as well.

*The nice thing about the structure of the trauma network is: you realize that everybody’s having the same problems, everybody’s looking for solutions, one is supportive of one another. (IP 18 l)*

Two interview partners, however, disagreed on the assertion that trust had developed within the group *(‘Calling it “trust” would be carrying things too far’*, IP 10 l).

By feeling included in the network, by exchanging facts about (common) patients and learning that other TNO members had similar problems, 21 out of 23 interview partners experienced a sense of togetherness within the network (‘*We trauma surgeons in Eastern Bavaria’*, IP 01 r), some members of the smaller hospitals even pride.

*Everyone is (…) a bit proud to be part of the network (…) You can say there’s a communal spirit existing [in the network]. Having common patients unites. (…) ‘Culture’ somehow also is the collective stock of stories that one shares with one another, and of course this grows, and consolidates. (IP 02 l)*

Two interview partners disagreed, mainly because they did not feel a need to get involved into the network.

*You can’t say that (…) the participating hospitals and surgeons are a die-hard team. (…) I don’t feel so ‘belonging’ that I need this ‘gang’ by all means. (IP 12 l)*

Some interviewees reported that they feel somewhat obliged to get actively involved in the TNO meetings, e.g. by presenting case studies, relating their experiences, or collecting data for studies. Two members described that they experienced a gentle social expectation (by the representative of the lead hospital, but also the other members) to communicate administrative as well as medical challenges, an expectation, however, which they did not take offense at, as they feel that this is the prerequisite for the beneficial functioning of the network.

*It’s expected that you report your practical experiences (…) and of course [experiences] from the auditing process, that you explain the problems which have come up, so another clinic which is still about to be audited … can consider it. (IP 10 l)*

4. Shared goals of the network relate to medical and political aspects

Optimizing the medical care of multiple trauma patients by including all stakeholders involved in the processes of care and by cooperating more smoothly could be identified as the main common goal, named by the majority of the members. Some explicitly referred to the targets that the German Trauma Society had set out in relation to implementing trauma networks.

*[We share], very plainly, the goal that one fulfills the requirements of the DGU [German Trauma Society], simply achieving a trauma care covering the whole area: within 20 minutes the accident patient is admitted to a trauma clinic, no matter where the accident has happened. Well, these are goals that we have already put into practice by now. (IP08 r)*

Some interviewees pointed out that pursuing the common goal of an improved patient care in everyday work was sometimes compromised by economic interests dominating the respective hospital management strategies. This mainly applied to the lack of resources.

*Practicing sensible medicine… simply depends on the structure of the respective hospitals, and this is something that the TNO cannot preset, of course. When the hospital administration isn’t willing to invest, or you don’t have enough colleagues available, then you can have the best of intentions to perform greatly, but it doesn’t work. (IP 09 r).*

In addition, some interview partners implied that the decisions to transfer (or not to transfer) a patient to another hospital was not always guided by medical reasons, but also by strategic or financial reasons.

*That business of transferring patients…well, these [accident] cases are talked about a lot, it’s in the papers, the media coverage is enormous, – and each senior consultant is duty-bound to their hospital administration, their commercial director, their county commissioner, you know? And then it goes: ‘Why did you have to transfer so many patients? Why couldn’t you do that on your own?’ (IP 15sr)*

One interview partner pointed out that the experience of clinical difficulties due to economic or administrative constraints was a factor binding the network members closer together.

*You get the feeling that you’re in the same boat, you’ve got the same problems: to secure the survival of traumatology [in one’s hospital] despite financial difficulties, covering the peaks of work load with limited staff and resources – we’re all comrades in suffering…the situation that the hospital administration prefers the elective surgery, and the unpredictable trauma surgery has to struggle…- This results in a feeling of togetherness. (IP 19l)*

Maintaining the number and constellation of participating hospitals in the TNO could also be identified as an important shared goal. During the time of the study, many hospitals were equally concerned with aggravations of the external regulations, e.g. increased certification requirements put forward by the German Trauma Society. The network members reported that these common concerns formed a bond between the respective hospitals’ representatives (mainly the local trauma centers). The novel regulations, e.g. requiring a 24 h-presence of anesthesiologists, were perceived as threatening the successful re-certification of some local centers. Fighting these new regulations turned out to be a shared interest of the TNO: For the local centers, the certification status was essential to remain part of the TNO, and was hence viewed to be of existential importance. The (supra)regional centers claimed that keeping the local centers within the TNO was crucial for a balanced distribution of workload across the region, but also crucial for the sake of the network idea.

*Although we’re not affected ourselves, we have to join the fight for the weakest ones in the network for the sake of the network idea, so they can stay [with the TNO] (…), so they can benefit from the network idea in a positive way. (IP 15 sr)*

5. Benefits on the network level: Communal spirit helps defend the network’s political interests in joint actions, and social relations enable smoother inter-hospital interactions

It was reported that as a consequence of the shared goal of maintaining the TNO in its current constellation, the group had advocated collectively for their interests, e.g. on conferences, with the trauma society, or with the municipality.

*When those local trauma centers were about not to be accredited [again], and the region-wide availability of health care wouldn’t have been ensured anymore, there were those letters to the county commissioner. (…) Evidently, we entered the stage as trauma network, we handed over the petition. That was an appearance of our trauma network. (IP 14 r)*

The group convinced the representative of the lead hospital to exert his influence on decision-makers. The group had been successful in so far that the new certification regulations would (for the time being) not apply to the TNO.

On the individual level, knowing the other TNO partners (better) and being able to appraise their specific expertise, e.g. by presented case studies, was the resource which was reported to be utilized most often in daily work (knowledge resource). The participating surgeons benefitted by this knowledge resource in different ways, which are detailed in Table 3. Above all, knowing each other better made co-operations between the hospitals more smoothly, which was perceived as a relief in the daily work.

**Table 3:** Benefits of knowing the other TNO members and of being able to appraise their expertise

	Benefit	Sample quote
1	Finding one’s bearings in the regional trauma care, especially valuable for newly appointed medical directors	- *I came ‘from the outside’, and I used to have few contacts only [in the region], didn’t know anybody in the [surgical] associations or panels of the region… And owing to the trauma network, our regular meetings, I got to know [the others] personally, and then you can catch up in other contexts. (IP 07 r)*
2	Making interactions easier and smoother, especially by knowing exactly whom to address in cases of questions and patient transfer	- *You gain more assurance when handling problematic cases, by not … having to think: ‘O, I have to phone with the big university. Who knows whom I get on the line there?’… Instead, you know the people, who are your contact persons there. … This is a priceless advantage. (IP 04 r)*
3	Being more confident and feeling safer when transferring patients to other hospitals	- *It’s good when you know, through the trauma network meetings: Okay, those guys can do it, I can’t, so I can hand the case over to them. And you don’t just send your patients someplace, but you know they’re in good hands. (IP 13 l)*
4	Increasing credibility among patients by being able to consult other trauma experts	- *We like to seek a second opinion (…) We are just a small rural hospital … And then you may have the critical patient and (…) a trauma that you don’t treat so often (…) (Then) it makes … a reasonable, a favorable impression that we don’t [say]: ‚Hurray, we know how to do everything!’ but: ‘Ok, we seek some advice.’ (IP 13 l)* - *There’s always those things where patients … say: ‘Doesn’t that have to be handled in a big center?’, and when you say: ‘We have sent your x-rays to this and that hospital, and the uni recommends [to treat you that way], and that’s exactly the way we would have done it, too’, − this is an incredible reassurance for the patients. (IP 07 r)*

When the surgeons explained the benefits of the network, the gain of medical knowledge played a minor role. Some interview partners appreciated the honest exchange of experience when handling certain trauma cases, but others also denied learning more in terms of clinical expertise.

*The ultimate goal is always the optimal care for the individual patient, and everything that’s not working out well on that track is clearly brought up. But without pointing the finger…, but: How can we do better? … How can we prevent those mistakes?... Otherwise, something like that could happen to us as well… So we’re learning, and our stock of knowledge has grown enormously through this cooperation (IP 15 sr).*

The majority of interview partners denied that being part of the TNO influenced their motivation to work or their job satisfaction; this would rather depend on factors related to the direct hospital environment.

*The trauma network has certainly contributed to the motivation, indirectly, by giving us the opportunity to engage in the care of multiple trauma patients. But I’d rather not say that there’s a direct motivation, in the way that I feel better when driving to work in the morning…here it’s the colleagues that render the work [enjoyable]. (IP12 l)*

6. Benefits on the hospital level (strategic benefits): Social capital is perceived to have only indirect effects on hospital performance, but can strengthen a team’s credibility

There are some hints that benefits of the trauma network’s social capital could also be achieved on the hospital level, i.e. by improving the preparations for the audit procedure, by improving conditions for clinical performance, and by increasing the hospital’s reputation. The (re-)auditing processes, which were critical for becoming and staying a member hospital of the trauma network, were perceived as challenging by many interview partners. Some surgeons pointed out that practical knowledge on how the audits could be successfully managed, and which pitfalls exist, was appreciated as a valuable benefit of the trauma network. Consequent changes in structure and processes were perceived to be beneficial as well.

*If you have a problem, then this is maybe communicated to all the others, as you need the information. This was what really happened in the beginning, someone has made this and that experience, he told the others and vice versa, this works out well […] The trauma network has certainly helped us rethink our structures, who is responsible et cetera… It’s interesting to learn from the others, the experiences they made at the audit and so on. (IP8 r)*

Few interview partners cautiously argued that the intensified personal contacts and the smoother interactions with other TNO hospitals could lower barriers in care within their “home” hospital, thus possibly improving clinical outcomes; consultations with surgeons from other hospitals can be initiated without restraints, the timeliness of patient care could be improved.

*You’re gaining some more confidence and you are acting safer in difficult cases, because you know the people [from the university hospital] who are your contact persons, you trust them …to tell you: “This and that’s what we would suggest to do.” (IP4 r)*

According to the interview partners, being part of a regional health care network also improved the hospital’s reputation in the local population, and increased the credibility among patients (see also Table 3).

## Discussion

### Principal findings

According to the surgeons’ accounts, the trauma network (TraumaNetzwerk DGU®) Eastern Bavaria (TNO) succeeded in becoming a group of trauma surgeons with a substantial bonding social capital. The inclusive but at times slightly demanding leadership style was considered important for establishing a certain norm of reciprocity in the network. At the same time, network members reported that empathy and respect by the TNO coordinator supported an atmosphere where TNO members felt confident to talk openly about their concerns, e.g. relating to re-certification processes. This resulted in a sense of community among the vast majority of the 23 interviewed members, and in joint endeavors to find solutions to collective problems. We could also identify a certain linking social capital; the hierarchy inherent in the network’s structure (representatives of three different levels of care) seemed to play only a minor role because the interactions were perceived to be respectful and appreciative. The TNO members individually benefitted from the social capital by being more confident in inter-hospital interactions. TNO members, especially of smaller hospitals, also felt they had a higher medical credibility among patients because they could rely on other TNO members in difficult trauma cases. The interviewees also described how social capital could be translated into power when the group successfully acted together in averting the certification authorities to exclude smaller TNO hospitals, and thus defended their status as a network. In addition, the interviews showed that the coordinator of the network could broker the groups concerns to external decision makers. We also found out on constraints to commitment and to social capital, since the network goals were perceived as being sometimes in tension with singular economic interests of member hospitals, and some respondents expressed that they did not feel the need to commit themselves strongly for various reasons.

### Strengths and weaknesses of the study

The analysis is restricted to a single German trauma network, so the results cannot be transferred to other trauma networks. In fact, we found that the leadership style was essential to create trustworthiness, communal spirit and reciprocity in the studied network, which implies that other trauma networks, whose coordinators may run the network in a different way, may most certainly have a different level of social capital. However, shedding light on the processes involved in generating a trauma network’s social climate and culture may help understand the dynamics of other trauma networks and inter-organizational networks as well. From a gendered perspective, the fact that all of the interviewees were male could also compromise the generalizability of the results. The predominantly male composition of trauma networks is a common finding in Germany, as only about 1% of German medical directors in the specialty of trauma surgery are female, and the official trauma network representatives of a hospital are the responsible (senior) consultants. Gender differences in consultants’ interactions with other health professionals have been described, e.g. regarding directive behavior or dominance [49], so the gender composition of a network may influence the way social capital is generated within the network. Studies on social capital from other disciplines imply that collaboration, solidarity, and reciprocity may increase in groups where women are present [50].

The development and utilization of social capital is a dynamic process, and probably it cannot be fully captured in a one-time cross-sectional data collection. Therefore, a longitudinal study may have produced richer results. However, we chose the trauma network with the longest history in Germany (certified in 2009), and due to its stable composition, the majority of its interviewed members related their experiences made over a period of about 5 years. This helped understand the interview partners’ view of a chronological sequence and causal relationship, respectively of conditions, activities, behaviors, and events.

Another limitation is that we failed to interview 3 out of 26 TNO members, who were probably less attached to the network than many of the other interviewees. Therefore, we might have missed some more critical perspectives on the network. We strove to compensate for this potential bias by giving special attention to statements that contradicted the emerging idea of social capital in the studied TNO and by incorporating these disconfirming data in the presentation, a process known as ‘deviant case analysis’ [48].

Despite the missing interviews, we consider it a strength of the study that we could base our analysis upon a broad data material of 23 out of 26 trauma network representatives. In addition, the data interpretation could be confirmed by respondent validation.

### Comparison with other studies

We could not identify empirical studies that analyzed the social texture and development of social capital within a professional network of medical peers. The literature on social capital in the health care industry largely consists of quantitative studies addressing individual benefits that networks can confer to participating health professionals; the networks studied were mostly teams in a singular healthcare institution. For example, high social capital in a hospital was shown to increase health professionals’ job satisfaction [34, 51] and to reduce their job tension in times of crisis [37]. Our interviews showed that being part of the trauma network did not explicitly influence the interviewees’ motivation to work; obviously, the positive social aspects related to the TNO membership were by far not as relevant for the surgeons’ everyday work as the direct workplace environment.

In addition, other studies have proposed that the transfer of medical knowledge is related to social capital [42, 43, 52] and a main benefit attributed to hospital alliances [9, 10]. Our study was not designed to analyze which characteristics of the network promoted or attenuated dissemination of knowledge. It became clear, however, that among the interviewed TNO members, the gain of technical information was of much less importance than the exchange of information on procedural and political aspects.

Our findings on the importance of leadership in the development of social capital confirm previous findings in the healthcare sector, but also in a variety of other organizational settings, e.g. by Hammer et al. [53] or Top et al. [54]. The majority of these studies use a quantitative research design proving an association between leadership style, work climate and social capital. Hence, our results help understand better the mechanisms of how exactly leadership may set a stage for the development of norms of reciprocity and mutual trust.

The leadership style in the trauma network helped develop a vertical dimension of social capital as well, when the TNO leader, being in a position of power in the wider trauma surgery community, advocated for the interests of the smaller hospitals among relevant decision-makers. This vertical dimension of social capital has been described in the political and developmental literature, e.g. when civic engagement succeeded in social change because their actions have been ‘scaled up’ through co-operations with more influential actors [30, 55].

### Major implications

Trauma networks have been installed worldwide with the aim of coordinating and improving patient care. Our study shows that apart from addressing quality of care, trauma networks may, by way of strengthening social capital among its members, serve as a valuable resource for the participating surgeons. The results also imply that some member hospitals could exploit the social capital for strategic benefits: The easy access to peers from the TNO was used to increase a surgical staff’s credibility among its patients, and the TNO’s collective activity protected some local hospitals from being excluded from the trauma network by the certification authorities. The latter example illustrates how access to power through social capital was used to secure a status and maintain a privilege. This phenomenon has been described for social capital and recognized as its potential downside, as in some cases, these privileges may be beneficial for one group, but potentially undesirable for society at large [27, 30]. It has already been described that hospital alliances can increase their political power through efforts such as lobbying [8]; however, this refers to official decisions and activities of the respective hospital administrations, whereas in our study, the political influence was reported to be exerted through the doctors themselves. This implies that political power in the healthcare setting cannot only be attained through the strategical policy of single allied hospitals, but also can also emanate from to the solidarity and social capital of a group of physicians.

It also became clear that trauma networks, by including hospitals of different levels of care, principally run the risk of a hierarchical structure where representatives of supra-regional hospitals are dominant. In the case of the Trauma Network Eastern Bavaria, the network members emphasized that an empathetic and respectful leadership style was key to making the representatives of the smaller hospitals feel included and appreciated. This was seen as key prerequisite for the development of trust and thus the vital source of social capital in this group. This finding also implies that medical professionals could benefit from advanced training on leadership skills in order to prepare them properly for key positions in the healthcare sector.

Within the network, the surgeons felt that high levels of mutual trust could be generated, which in turn facilitate cooperative behavior. Since cooperative behavior of physicians in charge is essential for effective inter-hospital patient care, networks such as the trauma network may be a useful organizational model for a variety of other healthcare challenges.

### Unanswered questions and future research

The study contributes to the understanding of how social capital can be generated in a regional network of German trauma surgeons, and how the group members can use this social capital for different ends. It would be interesting to compare and contrast these results with analyses of social capital in other trauma networks in Germany as well as in other European or North American countries.

Viewing the network functioning through a social capital lens entails a clear focus on resources based on social relations, and benefits derived from them. Potential disadvantages and challenges that arise when cooperating within a trauma network are only of marginal interest in this view, but are certainly present and may warrant a study in its own right.

In addition, one could focus on the aspect that physicians are usually affiliated with different other professional networks, e.g. federal or state wide medical associations, or networks of clinical directors [52]. Identification with and commitment in different professional networks and social settings might require the individuals to choose between at times conflicting goals, e.g. like the best possible trauma care for patients and economic interests of the home hospital. Our results have already hinted at that. Future research could show how these different collective goals are perceived and dealt with by professionals.

## Conclusion

Inter-hospital alliances are usually established with the aim of fostering efficiency and continuity of care and improving accessibility of patients. Our results shed a light on the fact that besides these strategic advantages, health professionals who are part of an inter-hospital network may also benefit individually from the co-operation, given there is a platform for regular exchange and interaction between hospital representatives. In the studied trauma network, this platform was the quarterly quality circle meeting, and participants perceived that the interactions and relationships that evolved in these meetings helped generate different forms of social capital. The results also highlight the important role of an empathic, respectful and inclusive leadership, otherwise the network may run the risk of strong hierarchical structures and dominances of certain representatives. As the number of inter-hospital alliances increases in many healthcare systems, it may be worth analyzing the respective co-operation processes, and defining structural and organizational prerequisites that are important from a health professional’s perspective. This study takes a first step in this direction, and can guide analyses in other contexts by providing a framework of relevant indicators.

## Abbreviation

TNO: Trauma Network Eastern Bavaria
